# Supplementary material for: Modeling glioblastoma heterogeneity as a dynamic network of cell states
Source: Mol Syst Biol. 2021 Sep 16;17(9):e10105. doi: 10.15252/msb.202010105 (PMC8444284; doi:10.15252/msb.202010105)
Supplement: Supplementary file 5 — Source Data for Figure 3 [file MSB-17-e10105-s001.zip › Figure3A_sourcedata/GSEA_3065/hallmarks_state2.GseaPreranked.1623416269049/HALLMARK_OXIDATIVE_PHOSPHORYLATION.html]

Details for gene set HALLMARK\_OXIDATIVE\_PHOSPHORYLATION[GSEA]

|  || Dataset | state2 |
| Phenotype | NoPhenotypeAvailable |
| Upregulated in class | na\_pos |
| GeneSet | HALLMARK\_OXIDATIVE\_PHOSPHORYLATION |
| Enrichment Score (ES) | 0.5928585 |
| Normalized Enrichment Score (NES) | 2.1085563 |
| Nominal p-value | 0.0 |
| FDR q-value | 0.0 |
| FWER p-Value | 0.0 |
Table: GSEA Results Summary

  

Fig 1: Enrichment plot: HALLMARK\_OXIDATIVE\_PHOSPHORYLATION      
 Profile of the Running ES Score & Positions of GeneSet Members on the Rank Ordered List

  

| PROBE | GENE SYMBOL | GENE\_TITLE | RANK IN GENE LIST | RANK METRIC SCORE | RUNNING ES | CORE ENRICHMENT || 1 | SLC25A5 |  |  | 168 | 0.299 | 0.0006 | Yes |
| 2 | CYCS |  |  | 207 | 0.279 | 0.0140 | Yes |
| 3 | IDH2 |  |  | 218 | 0.272 | 0.0300 | Yes |
| 4 | CYB5A |  |  | 233 | 0.261 | 0.0448 | Yes |
| 5 | POLR2F |  |  | 253 | 0.250 | 0.0585 | Yes |
| 6 | NDUFV2 |  |  | 307 | 0.230 | 0.0672 | Yes |
| 7 | COX5A |  |  | 308 | 0.229 | 0.0815 | Yes |
| 8 | NDUFA6 |  |  | 338 | 0.222 | 0.0923 | Yes |
| 9 | LDHB |  |  | 343 | 0.219 | 0.1056 | Yes |
| 10 | ATP5MC2 |  |  | 411 | 0.198 | 0.1108 | Yes |
| 11 | COX8A |  |  | 414 | 0.197 | 0.1229 | Yes |
| 12 | GPX4 |  |  | 425 | 0.194 | 0.1340 | Yes |
| 13 | COX17 |  |  | 445 | 0.191 | 0.1440 | Yes |
| 14 | UQCRQ |  |  | 501 | 0.181 | 0.1494 | Yes |
| 15 | LDHA |  |  | 506 | 0.179 | 0.1602 | Yes |
| 16 | CYC1 |  |  | 520 | 0.178 | 0.1699 | Yes |
| 17 | VDAC3 |  |  | 528 | 0.176 | 0.1803 | Yes |
| 18 | UQCRH |  |  | 535 | 0.175 | 0.1906 | Yes |
| 19 | TIMM13 |  |  | 574 | 0.167 | 0.1970 | Yes |
| 20 | UQCRFS1 |  |  | 585 | 0.165 | 0.2063 | Yes |
| 21 | ECI1 |  |  | 607 | 0.162 | 0.2142 | Yes |
| 22 | MDH2 |  |  | 615 | 0.161 | 0.2236 | Yes |
| 23 | MRPL11 |  |  | 619 | 0.160 | 0.2333 | Yes |
| 24 | TIMM10 |  |  | 639 | 0.158 | 0.2411 | Yes |
| 25 | ATP5F1D |  |  | 669 | 0.154 | 0.2477 | Yes |
| 26 | MRPS15 |  |  | 683 | 0.152 | 0.2558 | Yes |
| 27 | VDAC1 |  |  | 713 | 0.149 | 0.2620 | Yes |
| 28 | ACADM |  |  | 715 | 0.148 | 0.2712 | Yes |
| 29 | SLC25A11 |  |  | 730 | 0.146 | 0.2788 | Yes |
| 30 | NDUFV1 |  |  | 732 | 0.146 | 0.2879 | Yes |
| 31 | NDUFB6 |  |  | 749 | 0.144 | 0.2952 | Yes |
| 32 | NDUFS6 |  |  | 760 | 0.143 | 0.3031 | Yes |
| 33 | TIMM50 |  |  | 788 | 0.140 | 0.3089 | Yes |
| 34 | ATP5PF |  |  | 803 | 0.138 | 0.3160 | Yes |
| 35 | ATP5PO |  |  | 804 | 0.138 | 0.3247 | Yes |
| 36 | NDUFB2 |  |  | 832 | 0.134 | 0.3302 | Yes |
| 37 | PHB2 |  |  | 838 | 0.134 | 0.3381 | Yes |
| 38 | TOMM22 |  |  | 863 | 0.131 | 0.3437 | Yes |
| 39 | PRDX3 |  |  | 877 | 0.130 | 0.3505 | Yes |
| 40 | ATP5MG |  |  | 893 | 0.129 | 0.3570 | Yes |
| 41 | COX6B1 |  |  | 921 | 0.126 | 0.3619 | Yes |
| 42 | NDUFS8 |  |  | 931 | 0.125 | 0.3688 | Yes |
| 43 | ATP5MC1 |  |  | 935 | 0.125 | 0.3763 | Yes |
| 44 | UQCR10 |  |  | 941 | 0.125 | 0.3836 | Yes |
| 45 | MRPS11 |  |  | 953 | 0.123 | 0.3902 | Yes |
| 46 | TIMM8B |  |  | 955 | 0.123 | 0.3978 | Yes |
| 47 | NDUFAB1 |  |  | 970 | 0.122 | 0.4040 | Yes |
| 48 | IMMT |  |  | 988 | 0.121 | 0.4097 | Yes |
| 49 | ATP5PD |  |  | 994 | 0.120 | 0.4167 | Yes |
| 50 | HSPA9 |  |  | 996 | 0.120 | 0.4242 | Yes |
| 51 | NDUFB4 |  |  | 1027 | 0.117 | 0.4283 | Yes |
| 52 | COX7B |  |  | 1053 | 0.115 | 0.4328 | Yes |
| 53 | SUCLG1 |  |  | 1076 | 0.113 | 0.4375 | Yes |
| 54 | VDAC2 |  |  | 1083 | 0.112 | 0.4439 | Yes |
| 55 | MRPS12 |  |  | 1103 | 0.111 | 0.4488 | Yes |
| 56 | ATP5MF |  |  | 1132 | 0.109 | 0.4527 | Yes |
| 57 | HSD17B10 |  |  | 1143 | 0.109 | 0.4584 | Yes |
| 58 | FH |  |  | 1155 | 0.108 | 0.4640 | Yes |
| 59 | ATP5F1A |  |  | 1163 | 0.107 | 0.4699 | Yes |
| 60 | COX7C |  |  | 1181 | 0.106 | 0.4747 | Yes |
| 61 | NDUFC1 |  |  | 1187 | 0.105 | 0.4808 | Yes |
| 62 | MGST3 |  |  | 1210 | 0.104 | 0.4849 | Yes |
| 63 | ATP5F1C |  |  | 1211 | 0.104 | 0.4914 | Yes |
| 64 | FDX1 |  |  | 1224 | 0.103 | 0.4966 | Yes |
| 65 | NDUFA2 |  |  | 1244 | 0.102 | 0.5009 | Yes |
| 66 | ATP5F1B |  |  | 1269 | 0.100 | 0.5046 | Yes |
| 67 | NDUFS3 |  |  | 1288 | 0.099 | 0.5089 | Yes |
| 68 | ETFA |  |  | 1308 | 0.097 | 0.5129 | Yes |
| 69 | ECHS1 |  |  | 1322 | 0.097 | 0.5176 | Yes |
| 70 | ETFB |  |  | 1334 | 0.096 | 0.5224 | Yes |
| 71 | NDUFB8 |  |  | 1340 | 0.095 | 0.5279 | Yes |
| 72 | MTX2 |  |  | 1359 | 0.094 | 0.5318 | Yes |
| 73 | COX7A2 |  |  | 1407 | 0.092 | 0.5325 | Yes |
| 74 | SDHB |  |  | 1429 | 0.091 | 0.5360 | Yes |
| 75 | ATP5F1E |  |  | 1433 | 0.090 | 0.5413 | Yes |
| 76 | NDUFB3 |  |  | 1444 | 0.090 | 0.5459 | Yes |
| 77 | NDUFB1 |  |  | 1456 | 0.089 | 0.5503 | Yes |
| 78 | ATP6V1F |  |  | 1461 | 0.089 | 0.5554 | Yes |
| 79 | NDUFB7 |  |  | 1596 | 0.082 | 0.5461 | Yes |
| 80 | IDH3B |  |  | 1635 | 0.080 | 0.5470 | Yes |
| 81 | GRPEL1 |  |  | 1664 | 0.079 | 0.5489 | Yes |
| 82 | NQO2 |  |  | 1697 | 0.077 | 0.5503 | Yes |
| 83 | NDUFA3 |  |  | 1702 | 0.077 | 0.5547 | Yes |
| 84 | MRPL15 |  |  | 1714 | 0.076 | 0.5583 | Yes |
| 85 | TIMM9 |  |  | 1765 | 0.074 | 0.5575 | Yes |
| 86 | SLC25A3 |  |  | 1824 | 0.071 | 0.5557 | Yes |
| 87 | PDHB |  |  | 1825 | 0.071 | 0.5602 | Yes |
| 88 | UQCR11 |  |  | 1831 | 0.071 | 0.5641 | Yes |
| 89 | NDUFA8 |  |  | 1836 | 0.071 | 0.5681 | Yes |
| 90 | NDUFS4 |  |  | 1844 | 0.071 | 0.5718 | Yes |
| 91 | MDH1 |  |  | 1845 | 0.070 | 0.5762 | Yes |
| 92 | ATP5ME |  |  | 1867 | 0.070 | 0.5783 | Yes |
| 93 | MRPS22 |  |  | 1879 | 0.069 | 0.5815 | Yes |
| 94 | SLC25A6 |  |  | 1902 | 0.068 | 0.5834 | Yes |
| 95 | ACAA2 |  |  | 1924 | 0.067 | 0.5853 | Yes |
| 96 | MRPL34 |  |  | 1978 | 0.065 | 0.5837 | Yes |
| 97 | SDHD |  |  | 1999 | 0.065 | 0.5856 | Yes |
| 98 | MPC1 |  |  | 2005 | 0.064 | 0.5891 | Yes |
| 99 | AIFM1 |  |  | 2050 | 0.063 | 0.5883 | Yes |
| 100 | ATP5MC3 |  |  | 2068 | 0.062 | 0.5903 | Yes |
| 101 | COX5B |  |  | 2105 | 0.061 | 0.5903 | Yes |
| 102 | UQCRB |  |  | 2155 | 0.059 | 0.5887 | Yes |
| 103 | IDH3A |  |  | 2185 | 0.058 | 0.5892 | Yes |
| 104 | ATP6V1G1 |  |  | 2201 | 0.057 | 0.5912 | Yes |
| 105 | HTRA2 |  |  | 2236 | 0.056 | 0.5910 | Yes |
| 106 | CS |  |  | 2262 | 0.055 | 0.5917 | Yes |
| 107 | TIMM17A |  |  | 2284 | 0.054 | 0.5929 | Yes |
| 108 | GPI |  |  | 2330 | 0.053 | 0.5913 | No |
| 109 | UQCRC1 |  |  | 2355 | 0.052 | 0.5920 | No |
| 110 | NDUFA4 |  |  | 2462 | 0.049 | 0.5836 | No |
| 111 | ISCA1 |  |  | 2479 | 0.048 | 0.5849 | No |
| 112 | COX6C |  |  | 2515 | 0.047 | 0.5841 | No |
| 113 | OPA1 |  |  | 2557 | 0.046 | 0.5825 | No |
| 114 | TOMM70 |  |  | 2558 | 0.046 | 0.5854 | No |
| 115 | NDUFA7 |  |  | 2635 | 0.043 | 0.5799 | No |
| 116 | NDUFA5 |  |  | 2662 | 0.043 | 0.5798 | No |
| 117 | DECR1 |  |  | 2696 | 0.042 | 0.5788 | No |
| 118 | ACAT1 |  |  | 2710 | 0.041 | 0.5800 | No |
| 119 | ALAS1 |  |  | 2766 | 0.040 | 0.5766 | No |
| 120 | NDUFB5 |  |  | 2774 | 0.040 | 0.5783 | No |
| 121 | ATP5PB |  |  | 2782 | 0.039 | 0.5800 | No |
| 122 | DLD |  |  | 2865 | 0.037 | 0.5735 | No |
| 123 | ALDH6A1 |  |  | 2912 | 0.036 | 0.5708 | No |
| 124 | PDHA1 |  |  | 2916 | 0.036 | 0.5727 | No |
| 125 | COX7A2L |  |  | 2965 | 0.035 | 0.5697 | No |
| 126 | MRPL35 |  |  | 3072 | 0.032 | 0.5602 | No |
| 127 | DLAT |  |  | 3116 | 0.031 | 0.5576 | No |
| 128 | CASP7 |  |  | 3134 | 0.031 | 0.5577 | No |
| 129 | NDUFA1 |  |  | 3177 | 0.030 | 0.5550 | No |
| 130 | ATP6V1E1 |  |  | 3189 | 0.030 | 0.5557 | No |
| 131 | CYB5R3 |  |  | 3198 | 0.030 | 0.5567 | No |
| 132 | SDHA |  |  | 3219 | 0.029 | 0.5564 | No |
| 133 | NDUFS7 |  |  | 3229 | 0.029 | 0.5572 | No |
| 134 | NDUFS1 |  |  | 3393 | 0.026 | 0.5412 | No |
| 135 | NDUFS2 |  |  | 3438 | 0.025 | 0.5381 | No |
| 136 | SLC25A4 |  |  | 3452 | 0.025 | 0.5382 | No |
| 137 | FXN |  |  | 3584 | 0.022 | 0.5255 | No |
| 138 | HCCS |  |  | 3622 | 0.022 | 0.5228 | No |
| 139 | NDUFA9 |  |  | 3676 | 0.021 | 0.5184 | No |
| 140 | GLUD1 |  |  | 3694 | 0.021 | 0.5178 | No |
| 141 | OAT |  |  | 4004 | 0.015 | 0.4854 | No |
| 142 | ECH1 |  |  | 4011 | 0.015 | 0.4857 | No |
| 143 | UQCRC2 |  |  | 4064 | 0.014 | 0.4810 | No |
| 144 | MAOB |  |  | 4065 | 0.014 | 0.4819 | No |
| 145 | MTRR |  |  | 4108 | 0.014 | 0.4782 | No |
| 146 | IDH3G |  |  | 4210 | 0.012 | 0.4681 | No |
| 147 | SURF1 |  |  | 4221 | 0.012 | 0.4677 | No |
| 148 | COX10 |  |  | 4345 | 0.010 | 0.4550 | No |
| 149 | PDHX |  |  | 4385 | 0.009 | 0.4514 | No |
| 150 | NDUFC2 |  |  | 4392 | 0.009 | 0.4513 | No |
| 151 | LRPPRC |  |  | 4429 | 0.008 | 0.4479 | No |
| 152 | PHYH |  |  | 4479 | 0.008 | 0.4431 | No |
| 153 | AFG3L2 |  |  | 4488 | 0.007 | 0.4427 | No |
| 154 | BAX |  |  | 4506 | 0.007 | 0.4413 | No |
| 155 | RHOT1 |  |  | 4528 | 0.007 | 0.4395 | No |
| 156 | BDH2 |  |  | 4538 | 0.007 | 0.4389 | No |
| 157 | ATP6V1D |  |  | 4650 | 0.005 | 0.4272 | No |
| 158 | HADHB |  |  | 4810 | 0.003 | 0.4102 | No |
| 159 | ISCU |  |  | 4841 | 0.002 | 0.4071 | No |
| 160 | SUCLA2 |  |  | 4860 | 0.002 | 0.4052 | No |
| 161 | MRPS30 |  |  | 4878 | 0.001 | 0.4035 | No |
| 162 | DLST |  |  | 4897 | 0.001 | 0.4016 | No |
| 163 | ACADSB |  |  | 4949 | 0.000 | 0.3961 | No |
| 164 | OXA1L |  |  | 4978 | 0.000 | 0.3931 | No |
| 165 | OGDH |  |  | 5093 | -0.001 | 0.3809 | No |
| 166 | MFN2 |  |  | 5136 | -0.002 | 0.3765 | No |
| 167 | SDHC |  |  | 5266 | -0.004 | 0.3628 | No |
| 168 | GOT2 |  |  | 5473 | -0.008 | 0.3410 | No |
| 169 | COX15 |  |  | 5517 | -0.009 | 0.3369 | No |
| 170 | NNT |  |  | 5519 | -0.009 | 0.3374 | No |
| 171 | HADHA |  |  | 5542 | -0.009 | 0.3355 | No |
| 172 | ATP6V1C1 |  |  | 5554 | -0.009 | 0.3349 | No |
| 173 | PMPCA |  |  | 5650 | -0.011 | 0.3253 | No |
| 174 | ABCB7 |  |  | 5759 | -0.012 | 0.3144 | No |
| 175 | ACO2 |  |  | 6010 | -0.017 | 0.2885 | No |
| 176 | ACAA1 |  |  | 6129 | -0.019 | 0.2769 | No |
| 177 | IDH1 |  |  | 6208 | -0.020 | 0.2697 | No |
| 178 | SUPV3L1 |  |  | 6342 | -0.022 | 0.2567 | No |
| 179 | COX4I1 |  |  | 6705 | -0.030 | 0.2195 | No |
| 180 | ETFDH |  |  | 6820 | -0.032 | 0.2092 | No |
| 181 | COX11 |  |  | 7083 | -0.039 | 0.1832 | No |
| 182 | COX6A1 |  |  | 7383 | -0.047 | 0.1539 | No |
| 183 | ACADVL |  |  | 7628 | -0.054 | 0.1309 | No |
| 184 | ATP6V1H |  |  | 8025 | -0.068 | 0.0924 | No |
| 185 | RHOT2 |  |  | 8124 | -0.073 | 0.0863 | No |
| 186 | ATP1B1 |  |  | 8199 | -0.077 | 0.0832 | No |
| 187 | ATP6V0B |  |  | 8815 | -0.124 | 0.0245 | No |
| 188 | POR |  |  | 9033 | -0.155 | 0.0107 | No |
| 189 | PDP1 |  |  | 9089 | -0.164 | 0.0151 | No |
| 190 | ATP6AP1 |  |  | 9160 | -0.179 | 0.0187 | No |
| 191 | ATP6V0E1 |  |  | 9173 | -0.182 | 0.0289 | No |
Table: GSEA details [plain text format]

  

Fig 2: HALLMARK\_OXIDATIVE\_PHOSPHORYLATION: Random ES distribution      
 Gene set null distribution of ES for **HALLMARK\_OXIDATIVE\_PHOSPHORYLATION**
